# Liver stiffness measurement predicts the difficulty of Kasai procedure in biliary atresia: a single center retrospective analysis of 199 patients

**DOI:** 10.1186/s12887-019-1846-3

**Published:** 2019-11-29

**Authors:** Qiulong Shen, Yajun Chen, Chunhui Peng, Wenbo Pang, Zengmeng Wang, Dongyang Wu, Kai Wang, Xinjie Huang

**Affiliations:** Department of General Surgery, Beijing Children’s Hospital, Capital Medical University, National Center for Children’s Health, No.56 Nanlishi St, Xicheng District, Beijing, 100045 China

**Keywords:** Biliary atresia, Fibrous portal plate, Liver stiffness measurement, Kasai procedure

## Abstract

**Background:**

Kasai procedure is the standard initial treatment of infants with biliary atresia. The key to perform a successful surgery is to accurately remove the fibrous portal plate near the liver hilum. Yet how to estimate surgical difficulty pre-operatively remains unclear. This study aims to design an algorithm that predicts the difficulty of Kasai procedure using liver stiffness measurement (LSM).

**Methods:**

One hundred ninety-nine patients were included from April 2012 to December 2016. The patients were all surgically diagnosed with biliary atresia. Group A comprised of patients with porta hepatis retraction (the angle between the plane of the fibrous porta plate and the plane of the medial liver closest to the plate was equal to or smaller than 90°), group B comprised of patients without porta hepatis retraction (the angle between the plane of the fibrous porta plate and the plane of the medial liver closest to the plate was greater than 90°). Liver function measurements and LSM were measured for all patients within three days before surgery.

**Results:**

Our study included 19 cases in group A (9 males, 10 females) and 180 cases in group B (87 males, 93 females). LSM had statistical differences between the two groups, 28.10(14.90) kPa VS 10.89(7.10) kPa, *P* = 0.000. There was a significant relationship between LSM and operative age, TBA, AST, GGT (*P* = 0.000, 0.003, 0.003, 0.012, correlation coefficient = 0.323, 0.213, 0.207, 0.179). The AUROC of LSM was 0.919. When the cutoff value was 15.15 kPa(*OR* = 3.989; *P* = 0.000), the sensitivity, specificity, PPV, NPV and diagnostic accuracy were 0.947, 0.750, 0.285, 0.992 and 0.768, respectively. When the value was 23.75 kPa(*OR* = 3.483; *P* = 0.000), the sensitivity, specificity, PPV, NPV and diagnostic accuracy were 0.631, 0.950, 0.571, 0.960 and 0.919, respectively.

**Conclusions:**

LSM can be used to predict the difficulty in dissecting fibrous portal plate, and in turn, the difficulty of Kasai procedure. LSM > 23.75 kPa suggests a more complicated surgery.

## Background

Biliary atresia (BA) is an unique pediatric liver disease characterized by progressive inflammatory obliterative cholangiopathy. The Kasai procedure, introduced by Kasai from Japan, is the standard initial operation for treatment of infants with BA. The procedure aims to reconstruct biliary flow from the liver to the intestine. The adequate level of transection of the fibrous portal plate near the hilum of the liver is one of the most important steps, which is also the most difficult part of the operation. The adequate transection level of the fibrous portal plate plays a vital role in the early clearance of jaundice [1]. But there is no gold standard or descriptive articles that evaluate the difficulty of portal plate dissection. Surgical observation of our center shows that the porta hepatis retraction may affect the transection level owing to exposure difficulty. The porta hepatis retraction is more obvious in severe cirrhosis BA. LSM is a non-invasive and quantitative index that measures the degree of liver fibrosis. Therefore, the purpose of this study is to evaluate the porta hepatis retraction by preoperative non-invasive LSM examination, to assist in predicting the difficulty of the Kasai procedure.

## Methods

### Patients

Two hundred ninety-nine patients who were admitted to the General Surgery ward of Beijing Children’s Hospital during April 2012 to December 2016 underwent retrospective analysis. We selected the patients based on the following: 1. Diagnosed as biliary atresia during surgery, and also received Kasai surgery 2. Liver function measurements and LSM were obtained within 3 days before surgery. Liver function measurements concluded the alkaline phosphatase (ALP), alanine aminotransferase (ALT), aspartate aminotransferase (AST), total bilirubin (TBIL), direct bilirubin (DBIL), total bile acid (TBA) and r-glutamyl transferase (GGT). Thirty patients did not have pre-surgical LSM and were excluded from this study.

### Grouping method

We divided the patients into two groups based on surgical observation that was recorded at the time of surgery. Group A comprised of patients with porta hepatis retraction (the angle between the plane of the fibrous porta plate and the plane of the medial liver closest to the plate was equal to or smaller than 90°), group B comprised of patients without porta hepatis retraction (the angle between the plane of the fibrous porta plate and the plane of the medial liver closest to the plate was greater than 90°).

### LSM

Fibroscan (Echosens, France) was used to assess liver stiffness, and an experienced operator was responsible for obtaining the LSM. A probe (size S) was placed vertically on the skin surface between the right lower ribs. Ten values were then obtained avoiding major vessels. A median value calculated by the Statistics Analyze System was chosen as the final value, and the interquartile median ratio was less than 0.3.

### Statistical methods

The data were analyzed using the SPSS 19.0 statistical software. Two independent sample t-test and rank sum test were used to analyze the difference in the data between the two groups. Spearman correlation coefficient analysis was also used for data analysis. The receive operating characteristic curve (ROC curve) was used to determine the cutoff values. Logistic regression was used to assess the odds ratio (OR) and 95% confidence interval (CI). A *P* value < 0.05 was considered statistically significant.

## Results

### General characteristics

**Fig. 1:**
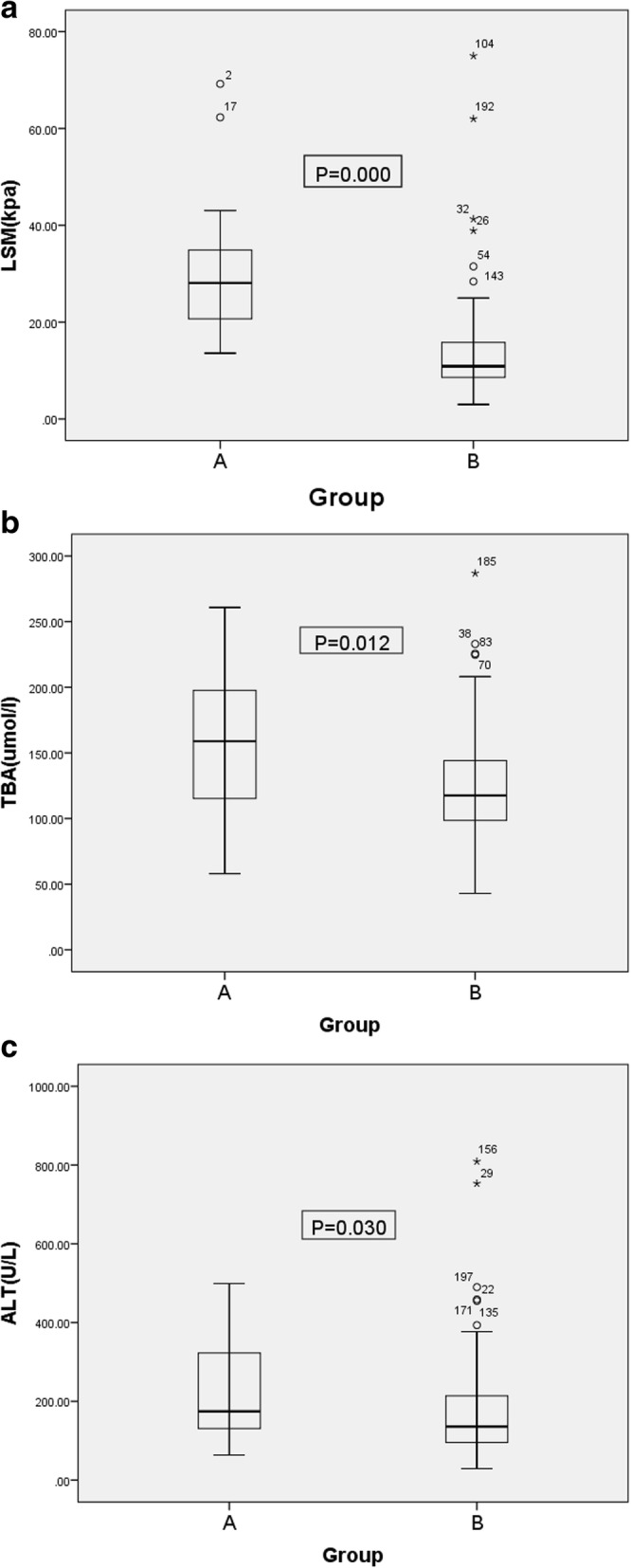
Box Plot of LSM(**a**), TBA(**b**), ALT(**c**)

One hundred ninety nine children (96 males and 103 females) with BA were included in this retrospective analysis. Among them, 19 cases were in group A(9 males, 10 females) and 180 cases were in group B(87 males, 93 females). Cases of group A(*n* = 19) had higher LSM, TBA, and ALT values than group B(*n* = 180; *P* = 0.000, 0.012, 0.030, respectively; Table 1, Fig. 1).

**Table 1:** General characteristics of two groups

	Group A(*n* = 19)	Group B(*n* = 180)	*P* value
LSM, median (IQR), kPa	28.10(14.90)	10.89(7.10)	0.000
TBIL, X ± S, umol/l	191.92 ± 69.63	188.83 ± 52.42	0.853
DBIL, X ± S, umol/l	95.75 ± 35.09	97.52 ± 25.78	0.833
TBA, X ± S, umol/l	156.53 ± 53.73	121.64 ± 38.44	0.012
GGT, median (IQR), U/L	401.00(729.50)	590.00(678.03)	0.459
ALT, median (IQR), U/L	174.40(219.80)	136.60(121.05)	0.030
AST, median (IQR), U/L	331.40(144.80)	261.75(155.98)	0.057
ALP, median (IQR), U/L	582.00(387.00)	568.00(317.00)	0.724
Operative Age, X ± S, d	82.47 ± 17.25	74.03 ± 21.01	0.059

*LSM* liver stiffness measurement; *TBIL* total bilirubin; *DBIL* direct bilirubin; *TBA* total bile acid; *GGT* Gamma-glutamyltransferase; *ALT* alanine aminotransferase; *AST* aspartate aminotransferase; *ALP* alkaline phosphatase

### Relationships between LSM and others

From the 199 cases, there was no relationship between LSM and ALP (*P* = 0.177), LSM and ALT (*P* = 0.058), LSM and TBIL (*P* = 0.188), or LSM and DBIL (*P* = 0.173). There was a significant relationship between LSM and operative age (*P* = 0.000, correlation coefficient = 0.323), LSM and AST (*P* = 0.003, correlation coefficient = 0.207), LSM and GGT (*P* = 0.012, correlation coefficient = 0.179), LSM and TBA (*P* = 0.003, correlation coefficient = 0.213) in the 199 cases; however, the correlation coefficients were not high.

### Cut-off value for the two groups

**Fig. 2:**
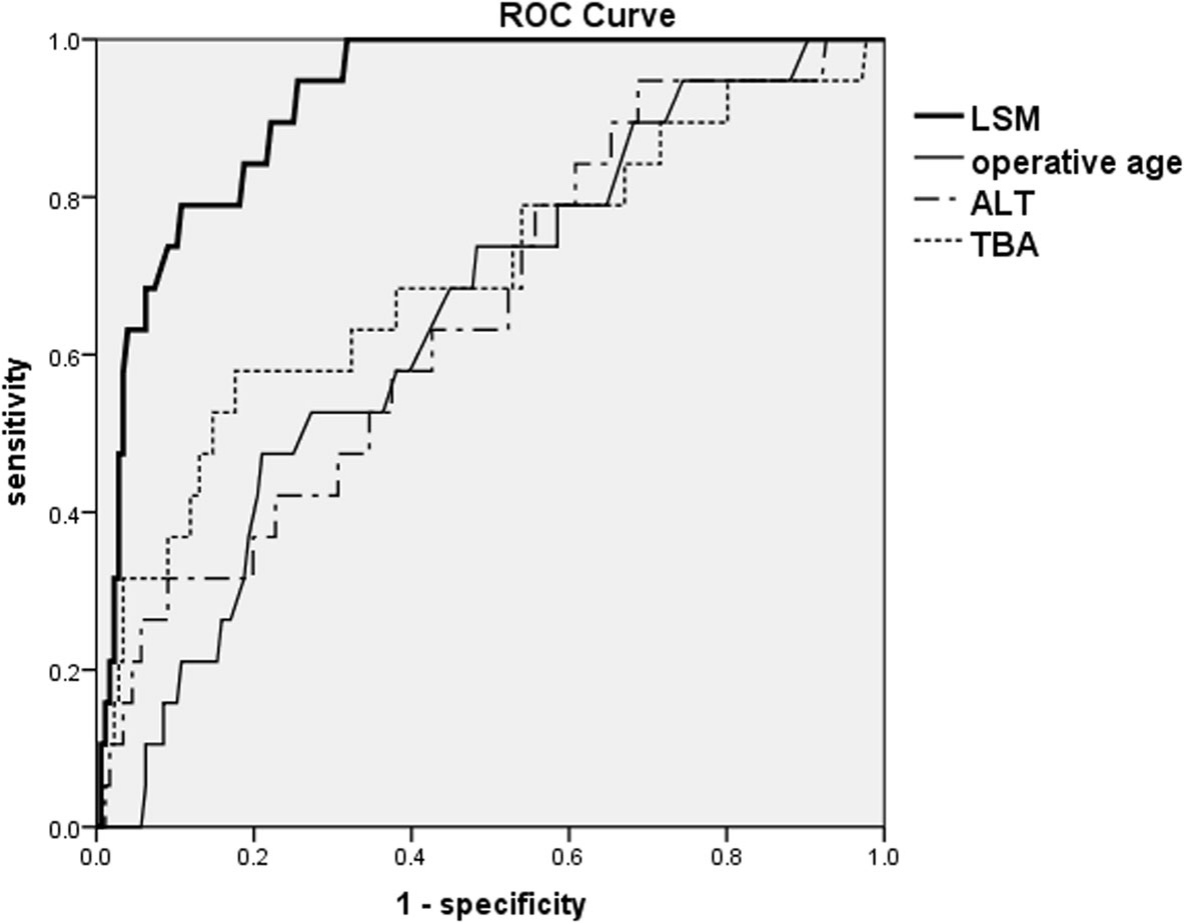
ROC curve of LSM, Operative age, ALT and TBA

The ROC curves of LSM, biochemical markers and the operative age were analyzed. The results of ROC analysis are shown in Table 2. *P* values of LSM, TBA, ALT and operative age revealed statistical significance (*P* = 0.000, 0.005, 0.031 and 0.038, respectively). The AUROC of LSM, TBA, ALT, and the operative age were 0.919, 0.698, 0.651and 0.645, respectively (Fig. 2). The AUROC of AST, TBIL, DBIL, and GGT were 0.633, 0.499, 0.479 and 0.444, respectively (*P* = 0.057, 0.990, 0.763 and 0.425, respectively).

**Table 2:** ROC Analysis Results of LSM, Biochemical Markers and the Operative Age

	AUROC	P value	Cutoff value	sensitivity	specificity
LSM, kPa	0.919	0.000	15.15	0.947	0.739
Operative age, d	0.645	0.038	87.0	0.474	0.790
ALT, U/L	0.651	0.031	109.45	0.947	0.313
TBA, umol/l	0.698	0.005	155.40	0.579	0.824
ALP, U/L	0.525	0.724	–	–	–
AST, U/L	0.633	0.057	–	–	–
GGT, U/L	0.444	0.425	–	–	–
TBIL, umol/l	0.499	0.990	–	–	–
DBIL, umol/l	0.479	0.763	–	–	–

### Efficiency evaluation of LSM in predicting the difficulty of the Kasai procedure

Only the AUROC of LSM was more than 0.7. Using the Jordan index, the optimal cut-off value of LSM was 15.15kpa, with a sensitivity of 0.947 and a specificity of 0.739. When the specificity was increased to 0.90, the cut-off value was 20.9 kPa and the sensitivity was low. When the specificity was increased to 0.95, the cut-off value was 23.75 kPa and the sensitivity was lower (Table 3).

**Table 3:** Efficiency Evaluation of LSM in Assessing the Difficulty of the Fibrous Plate Dissection of BA

	OR(95%CI)	P value	NPV	PPV	Sensitivity	specificity	Diagnostic accuracy
LSM > 15.15kpa versus≤15.15 kpa	3.989(7.009–416.015)	0.000	0.992	0.285	0.947	0.75	0.768
LSM > 20.9kpa versus≤20.9 kpa	3.290(8.614–83.673)	0.000	0.964	0.451	0.736	0.905	0.889
LSM > 23.75kpa versus≤23.75 kpa	3.483(10.333–102.674)	0.000	0.960	0.571	0.631	0.950	0.919

A LSM > 15.15 kPa, LSM > 20.90 kPa and LSM > 23.75 kPa were predictive of the difficulty of the Kasai procedure in univariate logistic regression analyses (OR = 3.989, 3.290 and 3.483; *P* = 0.000, 0.000 and 0.000, respectively). The positive and negative predictive values (PPV and NPV) of an LSM value cut-off > 15.15 kPa, 20.90 kPa and 23.75 kPa were 0.285, 0.451, 0.571 and 0.992, 0.964, 0.960, respectively. The diagnostic accuracies were 0.768, 0.889 and 0.919, respectively (Table 3).

## Discussion

BA is a unique pediatric liver disease characterized by progressive inflammatory obliterative cholangiopathy. If left untreated, fibrosclerosing obliteration progresses in both intrahepatic and extrahepatic bile ducts, which inevitably leads to liver cirrhosis [2]. The incidence of BA in Asia is reported to be as high as approximately 1 in 5000 live births [3].

Kasai procedure is a standard surgical treatment for BA, which has been widely carried out. The adequate level of transection of the fibrous portal plate is one of the most important steps in the Kasai procedure, which is also the most difficult part of the operation. The native liver survival (NLS) rate is clearly different in different centers [4–7].At present, most studies showed that early clearance of jaundice after Kasai procedure is an important factor for good prognosis [4, 7]. The adequate level of transection of the fibrous portal plate plays a key role in the early clearance of jaundice [1]. The more severe the degree of liver cirrhosis, the more difficult to anatomize the fibrous plate will be, which may lead to the transection level not exact enough. It can affect bile drainage and ultimately the clearance of jaundice.

There is no literature that describes exactly how to define the difficulty of dissecting the fibrous portal plate. A recent study has demonstrated that removing a part of the medial liver closest to the fibrous porta plate aids in exposure of the porta hepatis and provides the surgeon with a better surgical field [8]. Similar to the study, our center has found that more severe liver cirrhosis presents with more severe edema, which presents as porta hepatis retraction. This causes difficulty when exposing the fibrous portal plate during surgery and blocks the surgical field to varying degrees.

The Kasai procedure has been carried out in our center for more than ten years. The five-year NLS rate is 58% [9]. Through observation, we found that the more severe the cirrhosis, the more obvious the vascular proliferation around the fibrous portal plate, which led to easy bleeding during the dissection. If hemostasis is done by electrocoagulation, it will easily damage the fine ductules in the porta hepatis and this will affect prognosis. At the point of surgery, we had no means to measure vascular proliferation and this remains an ongoing challenge. A method to measure vascular proliferation would provide more insight to the degree of surgical difficulty.

The gold standard to judge the degree of liver fibrosis is liver biopsy, but it is an invasive examination. Before the operation, the degree of liver fibrosis of BA is judged only by clinical presentation, for example, operative age or blood test. But there is no uniform standard for the specific cut-off value, and there is still controversy surrounding the correlation between the operative age and the prognosis. A multivariable analysis including 244 case in a single center of China showed that the low 5 year NLS rate of children with BA was associated with the operative age over 90 days [10]. Moreover, other studies suggested that when the operative age was over 60 days, postoperative NLS rate declined and liver transplantation rate increased [11, 12]. However, several studies suggested that there is no correlation between the operative age and postoperative NLS [13, 14]. Studies have shown that 5 years and 10 years NLS rate of the children whose operative ages were over 100 days can reach 40 and 45% [15]. In this study, there was no correlation between the operative age in the two groups. Therefore, we speculated that the operative age might not be an effective index for judging severe cirrhosis and is not a factor for the difficulty of the surgical procedure.

LSM is a noninvasive technique which is used to assess the degree of liver fibrosis. The basic principle of LSM involves a one-dimensional transient elastographic wave, which has a distinguishable traveling speed in different media, and can be translated into various degrees of fibrosis. When the liver is hard, the transient elastographic wave travels faster, resulting in a higher LSM value (kPa). It has the characteristics of being noninvasive, accurate, rapid and repeatable. It has been widely used in the determination of liver fibrosis in adults [16–19], but the application of LSM in children is markedly fewer than in adults. It can be used to evaluate the degree of liver fibrosis in different liver diseases, auxiliary diagnosis of portal hypertension and upper gastrointestinal tract varices [20, 21]. The normal LSM of children is about 5.0 kPa, which is consistent with adults [22, 23]. LSM is a routine procedure at our hospital, and all patients with BA are sent for LSM before and after surgery, as well as during follow ups. The preliminary result of our center shows that LSM can be used to measure the degree of liver fibrosis in children with BA, and the cut-off value of liver cirrhosis is 15.15 kPa [24].

In this study, 199 cases of BA were reviewed and analyzed by ROC curve analysis. The cut-off value of LSM used to distinguish the two groups was 15.15kpa, and its sensitivity was high and the specificity was relatively low. When the cut-off value of LSM is 23.75 kPa, the sensitivity is relatively low and the specificity is significantly higher, which is consistent with the clinical observation of our center. According to our center’s early findings, BA children with an LSM less than 15.15 kPa did not reach liver cirrhosis [24]. Combined with the results of this study, when LSM was less than 15.15 kPa, there was little possibility of porta hepatis retraction. If LSM was greater than 23.75 kPa before operation, the possibility of porta hepatis retraction is high.

Studies have shown that LSM is influenced by aminotransferase, bilirubin and other factors [25–27]. In this study, ALP, ALT, AST, TBIL, DBIL, TBA and GGT were compared in the two groups. LSM was positively correlated with AST, GGT and TBA. But the correlation coefficient is not high.

There is no difference in the prognosis between BA with and without a Kasai procedure prior to liver transplantation [28, 29]. Although the incidence of complications increases, the Kasai procedure with effective bile drainage could significantly improve the liver transplantation-free survival [30], reduce the high demand for liver donors, and also effectively reduce the difficulty of liver transplantation. The results showed that when LSM reached a certain degree, porta hepatis retraction was notably present, portal plate dissection was relatively difficult, and the need to have the operation performed by a team with rich experience in the Kasai procedure was necessary. This was to ensure that bile drainage successfully occurred after the operation and to achieve long-term liver transplantation-free survival. Therefore, if conditions permit, BA children with LSM over 23.75 kPa should be transferred to a large clinical center with vast experience with the Kasai procedure for treatment, allowing for a greater chance of surgical success, and thus avoiding a meaningless operation and reducing the financial burden of the family.

## Conclusion

LSM can be used to predict the difficulty in dissecting fibrous portal plate, and in turn the difficulty of Kasai procedure. LSM > 23.75 kPa suggests a more complicated surgery.

## Data Availability

The data is available from the corresponding author on reasonable request.

## Abbreviations

ALP: Alkaline phosphatase
ALT: Alanine aminotransferase
AST: Aspartate aminotransferase
AUROC: Area under the receiver operating characteristic
BA: Biliary atresia
DBIL: Direct bilirubin
GGT: R-glutamyl transferase
LSM: Liver stiffness measurement
NPV: Negative predictive value
PPV: Positive predictive value
TBA: Total bile acid
TBIL: Total bilirubin
